# Immunomodulatory effect of *Lacticaseibacillus rhamnosus* and *Lactobacillus acidophilus* in human dendritic cells

**DOI:** 10.1007/s00203-026-05135-7

**Published:** 2026-08-10

**Authors:** Glauber Campos Vale, Ellen Sayuri Ando-Suguimoto, Karin Hitomi Ishikawa, Marcia Pinto Alves Mayer

**Affiliations:** 1 Department of Restorative Dentistry, Federal University of Piauí, Campus Universitário Ministro Petrônio Portella - Bairro Ininga, Terezina, 64049-550 Brazil; 2 Department of Microbiology, Institute of Biomedical Sciences, University of São Paulo, São Paulo, Brazil

**Keywords:** Probiotics, Cytokines, Gene expression, Inflammation and innate immunity

## Abstract

This study explored the immunomodulatory properties of the probiotic strains *Lacticaseibacillus rhamnosus* LR-32 and *Lactobacillus acidophilus* LA-5 by evaluating their effects on human dendritic cell (DC) responses. Immature DCs were obtained from human CD14⁺ monocytes and challenged with *E. coli* LPS and/or probiotic lactobacilli at a DC ratio of 1:10. Relative transcription of inflammatory mediators and pattern-recognition receptors was assessed by RT-qPCR. Cytokine production and surface marker expression were evaluated by ELISA and flow cytometry, respectively. DC function was further assessed after interaction with CD4⁺ T lymphocytes, followed by analysis of T cell phenotype by flow cytometry and cytokine production by ELISA. LPS upregulated the transcription of intracellular receptors AIM2, NOD1, and NOD2 and increased COX-2, TNF-α, IL-1β, IL-6, CXCL-8, IL-12, and IL-18 mRNA levels compared with immature DCs (*p* < 0.001). Exposure to probiotics downregulated most genes encoding receptors and inflammatory mediators compared with LPS-stimulated DCs. In LPS-stimulated DCs, LA-5 reduced intra- and extracellular receptor gene expression, increased COX-2, TNF-α, IL-1β, IL-6, CXCL-8, and IL-10 transcription, and decreased IL-12 mRNA levels. LR-32 upregulated TLR2, COX-2, IL-6, CXCL-8, and IL-10, while reducing IL-12 expression, although these effects were less pronounced than those induced by LA-5. LA-5-exposed DCs induced a higher frequency of CD25highFOXP3highCD127low CD4⁺ T cells than LR-32. These findings indicate that LA-5 and LR-32 modulate human DC responses in a strain-dependent manner. Rather than acting as broadly anti-inflammatory stimuli, these probiotics reshaped LPS-induced activation by modulating pattern-recognition receptor pathways, cytokine responses, and a Treg-associated CD4⁺ T cell phenotype, suggesting a potential role in controlled mucosal immune regulation.

**Supplementary Information:**

The online version contains supplementary material available at https://doi.org/10.1007/s00203-026-05135-7.

## Introduction

Periodontitis is a chronic inflammatory disease associated with a dysbiotic oral microbiota and an unbalanced host immune response (Darveau 2009; Hajishengallis and Lamont 2012). Periodontal tissue destruction is not determined exclusively by microbial challenge, but also by host-related mechanisms that regulate the magnitude and persistence of inflammation (Kinane et al. 2012). The innate immune system constitutes the first line of defense against periodontal microorganisms and includes epithelial barriers, soluble mediators, neutrophils, macrophages, and dendritic cells (DCs). These components interact with the complex microbial communities present in the oral cavity and contribute to the transition from innate recognition to adaptive immune responses (Paster et al. 2006; Wilensky et al. 2014).

DCs are professional antigen-presenting cells that play a central role in mucosal immune surveillance. In periodontal tissues, they recognize microbial-associated molecular patterns (MAMPs) derived from commensals, pathobionts, and periodontal pathogens through pattern-recognition receptors, including Toll-like receptors (TLRs), nucleotide-binding oligomerization domain-like receptors (NOD-like receptors), and inflammasome-related sensors (Hans and Hans 2011; Wilensky et al. 2014). Activation of these receptors triggers intracellular signaling pathways that regulate the expression of co-stimulatory molecules and inflammatory mediators, thereby influencing T cell polarization and the balance between protective immunity, chronic inflammation, and immune tolerance (Kapsenberg 2003; Song et al. 2018).

Periodontal pathobionts can interact with DCs through different microbial structures and may induce distinct immunological outcomes. For example, *Porphyromonas gingivalis* expresses virulence factors capable of altering immune surveillance and modulating DC function. Its LPS can preferentially activate TLR2-related pathways and induce a distinct maturation profile in human DCs, characterized by reduced stimulatory capacity (Kanaya et al. 2009). In contrast, LPS derived from *Escherichia coli* is a classical TLR4-dependent stimulus that promotes DC activation and favors inflammatory and Th1-associated responses (Pulendran et al. 2001). Therefore, although *E. coli* LPS does not reproduce the complexity of periodontal pathogens or polymicrobial oral biofilms, it provides a standardized and well-characterized inflammatory stimulus to investigate how probiotic strains modulate DC activation under controlled in vitro conditions.

DC function in periodontal tissues is also influenced by the surrounding mucosal microenvironment. Gingival epithelial cells, fibroblasts, and other resident cells can respond to microbial challenge by producing cytokines and chemokines that further shape DC activity (Eskan et al. 2008). Moreover, DCs exposed to soluble mediators produced by tissue cells may induce T helper 2 and regulatory T cell responses, whereas direct interaction between DCs and bacteria may favor T helper 1 differentiation (Rimoldi et al. 2005).Gingival epithelial cells and DCs also coordinate their responses to oral bacteria through the production of β-defensins, cytokines, and chemokines in a pathogen-specific manner (Yin et al. 2010). Thus, DCs are not only microbial sensors, but also key regulators of the quality and direction of adaptive immune responses in mucosal inflammatory diseases.

Probiotics have emerged as potential adjunctive approaches for the control of periodontal inflammation because they may interfere with pathogenic microorganisms, modulate biofilm ecology, reinforce epithelial barrier function, and regulate host immune responses. Lactobacilli, in particular, have been associated with maintenance of mucosal homeostasis (van Baarlen et al. 2013; Linares et al. 2017), partly due to their ability to modulate inflammatory and regulatory immune pathways under physiological and pathological conditions (Saarela et al. 2000; Boirivant and Strober 2007; Vissers et al. 2010; Bron et al. 2011). However, probiotic effects are highly strain-dependent, and the same bacterial genus or species may induce distinct host responses depending on the cell type, inflammatory context, bacterial dose, and time of exposure (Foligne et al. 2007; Mileti et al. 2009; Rask et al. 2013).

The strains *Lactobacillus acidophilus* LA-5 and *Lacticaseibacillus rhamnosus* LR-32 were selected because previous studies have indicated their potential to interfere with periodontal pathogens and modulate host-cell responses. These strains were shown to reduce adhesion and invasion of *P. gingivalis* in gingival epithelial cells and to decrease its abundance in multispecies biofilms while favoring commensal streptococci (Albuquerque-Souza et al. 2019; Ishikawa et al. 2020). In addition, studies using monocytes, epithelial cells, and dendritic cells have suggested that LA-5 and LR-32 do not elicit identical immunologic profiles, underscoring the need to investigate their strain-specific effects on antigen-presenting cells (Vale and Mayer 2021; Bueno et al. 2022; Vale et al. 2023).

One possible mechanism by which probiotics influence mucosal immunity is through the modulation of DC phenotype and function. DCs can recognize lactobacilli-derived structures through receptors such as TLRs and DC-SIGN, leading to changes in cytokine production, co-stimulatory molecule expression, and T cell polarization (Smits et al. 2005; Niers et al. 2005; Konstantinov et al. 2008). Certain lactobacilli can induce IL-10-producing regulatory T cells through modulation of DC function, whereas others may enhance inflammatory cytokine production or promote partial DC activation (Mohamadzadeh et al. 2005; Smits et al. 2005; Kwon et al. 2010). In mouse DCs, lactobacilli were also shown to modify LPS-induced expression of maturation markers and shift cytokine profiles toward a more regulatory or anti-inflammatory pattern (Luongo et al. 2017). These findings indicate that probiotic–DC interactions may result in either activating or regulatory outcomes, depending on the strain and inflammatory microenvironment.

Based on this background, the present study tested the hypothesis that *L. acidophilus* LA-5 and *L. rhamnosus* LR-32 modulate human monocyte-derived DC responses in a strain-dependent manner, particularly under LPS-induced inflammatory stimulation. To address this hypothesis, we evaluated DC viability, pattern-recognition receptor gene expression, inflammatory and regulatory mediator expression, cytokine secretion, surface marker expression, and the ability of probiotic-exposed DCs to influence a regulatory T cell-associated phenotype in CD4⁺ T cells. This approach was designed to clarify how these probiotic strains reshape DC responses under basal and standardized inflammatory conditions, while recognizing that direct extrapolation to periodontal disease requires future studies using oral pathobionts, pathogen-derived LPS, or multispecies periodontal biofilms.

## Materials and methods

### Ethics approval

The study protocol was reviewed and approved by the Research Ethics Committee of the Institute of Biomedical Sciences, University of São Paulo (protocol no. 2.556.792). All procedures complied with institutional and national ethical guidelines and were conducted in accordance with the Declaration of Helsinki and its subsequent revisions.

## Bacterial strains and growth conditions

Two commercially available probiotic strains were investigated: *Lacticaseibacillus rhamnosus* LR-32™ (Danisco, Madison, WI, USA) and *Lactobacillus acidophilus* LA-5™ (Chr. Hansen, Hørsholm, Denmark). Bacteria were cultured in de Man, Rogosa and Sharpe (MRS) broth and agar (Difco Laboratories, Detroit, MI, USA) under microaerophilic conditions (5% CO₂) at 37 °C. After growth to the mid-exponential phase, bacterial suspensions were standardized to approximately 2.0 × 10⁸ CFU/mL. and used in subsequent co-culture experiments. Bacterial counts were determined by OD600 using a previously established growth curve and confirmed by CFU counts. Plates were incubated under microaerophilic conditions at 37 °C for 48 h before colony counting.

## Generation of immature dendritic cells from cD14 + Monocytes

Peripheral blood mononuclear cells (PBMCs) were obtained from eight healthy donors (mean age 35 ± 5 years; six females and two males) by density gradient centrifugation using Ficoll (GE Healthcare, Logan, UT, USA). After isolation, PBMCs from all donors were pooled and cryopreserved in fetal bovine serum containing 10% dimethyl sulfoxide (DMSO) in cryogenic tubes at approximately 5 × 10⁶ cells/mL and stored at − 80 °C until use. Three independent experiments were performed on subsequent days. For each experiment, one cryotube from the pooled PBMC preparation was thawed and used for CD14⁺ monocyte isolation. CD14⁺ monocytes were isolated by magnetic-activated cell sorting (MACS; Miltenyi Biotec, Auburn, CA, USA), following the manufacturer’s instructions. Enriched monocytes (1.5 × 10⁶ cells/mL) were cultured in 24-well plates containing complete RPMI-1640 medium (GE Healthcare) supplemented with 10% heat-inactivated fetal bovine serum (Gibco-Invitrogen, Waltham, MA, USA), 1% l-glutamine (GE Healthcare), 1% penicillin–streptomycin (Thermo Scientific, Waltham, MA, USA), 2% sodium pyruvate (Gibco-Invitrogen, Waltham, MA, USA), 2% HEPES buffer (GE Healthcare), and 50 µM β-mercaptoethanol (Sigma-Aldrich, St Louis, MO, USA). Differentiation into dendritic cells was induced by the addition of recombinant human GM-CSF (70 ng/mL) and IL-4 (35 ng/mL) (MiltenyiBiotec, Auburn, CA, USA). Culture media were replenished on days 3 and 5 with fresh cytokine-supplemented medium.

## Interaction of probiotic with imature and LPS-treated dendritic cells

After 7 days, immature dendritic cells were harvested, washed, and resuspended at 2 × 10⁵ cells/mL in antibiotic-free RPMI medium. Probiotic bacteria were added at a DC: bacteria ratio of 1:10, in the presence or absence of lipopolysaccharide (LPS; 1 µg/mL, *E. coli* O111:B4, Sigma-Aldrich). Cells treated with LPS alone or left untreated served as positive and negative controls, respectively. Following 24 h incubation, culture supernatants were collected for cytokine analysis. Non -adherent bacteria were removed by washing the cells with PBS. Cell viability was determined. For gene expression analysis, 200 µl of RLT buffer (QIAGEN, Valencia, CA, USA) was added into each well and the cells detached and stored for further RNA extraction. DCs were also analyzed by flow cytometry to determine the expression of DC markers, using fluorescent antibodies. DCs function was evaluated after interaction with T lymphocytes differentiation, T cells phenotypes were determined by flow cytometry analysis, and cytokines production by ELISA.

## Cell viability assay

Cell viability was assessed indirectly by measuring lactate dehydrogenase (LDH) release into culture supernatants using the Pierce LDH Cytotoxicity Assay Kit (Thermo Scientific, Waltham, MA, USA), according to the manufacturer’s instructions. After 24 h of stimulation with LPS and/or probiotic strains, culture plates were centrifuged at 250 × g for 3 min, and 50 µL of each supernatant was transferred to a 96-well plate. Reaction mixture was added, and plates were incubated for 30 min at room temperature protected from light. The reaction was stopped, and absorbance was measured at 490 nm and 680 nm. LDH activity was calculated by subtracting the 680 nm background absorbance from the 490 nm reading. Spontaneous LDH release, maximum LDH release, and culture medium background controls were included. Cytotoxicity was calculated relative to spontaneous and maximum LDH release controls, and cell viability was expressed as 100 − % cytotoxicity.

### Gene expression (RT-qPCR)

Total RNA was extracted using the RNeasy Kit (QIAGEN, Valencia, CA, United States) after cell lysis, and its concentration and purity were determined spectrophotometrically at 260 and 280 nm in NanoDrop™ One Spectrophotometer (Thermo Scientific, Waltham, MA, United States). Complementary DNA (cDNA) was synthesized using SuperScript VILO Master Mix (Invitrogen, Waltham, MA, USA). Quantitative PCR reactions were performed using TaqMan^®^ Gene Expression Master Mix and specific probes targeting genes related to innate immune signaling and inflammation, including NLRP3, NOD1, NOD2, TLR2, TLR4, AIM2, COX-2, TNF-α, IL-1β, IL-6, CXCL-8, IL-10, IL-12, and IL-18. GAPDH was used as the endogenous control. Amplification was carried out in a StepOnePlus™ system (Applied Biosystems, Foster City, CA, United States) under standard cycling conditions using StepOnePlus™ System (Applied Biosciences, Foster City, CA, USA). Relative gene expression was calculated using the ΔΔCt method (Pfaffl 2001), using GAPDH as an endogenous control.

## Differentiation of CD4 + T lymphocytes and interaction with dendritic cells

CD4 + T cells were isolated from PBMCs by negative selection using the Human CD4 + T Cell Enrichment Kit (EasySep, Stem Cell Technologies Inc.), following manufacturer’s instructions, and then naive CD45RA + T cells were separated after depletion of CD45RO+ cells (Miltenyi Biotec GmbH, Bergisch Gladbach, Germany). Monocyte-derived DCs maturated after 24 h treatment with LPS and/or with probiotics were co-cultured with purified allogeneic CD4 + CD45RA + T cells in 48-well plates at DC: T-cell ratio of 1:10. After 5 and 8 days of incubation, cells were expanded with IL-2 (30 U). After 12 days, culture supernatants were collected for cytokine measurements, and cells were used to analyze the Treg phenotype by flow cytometry.

## Cytokines production

Cytokine levels in culture supernatants (DCs assays and B-lymphocytes) were measured using enzyme-linked immunosorbent assay (ELISA) kits (BD OptEIA™, BD Biosciences, CA, United States), following the manufacturer’s protocols.

### Flow cytometric analysis

Phenotypic studies of DCs and T lymphocytes were performed after staining with the appropriate monoclonal antibody using a FACSCanto II flow cytometer (Becton Dickinson, BD Biosciences, San Diego, CA). DCs were stained with anti-CD86 fluorescein isothiocyanate (FITC), CD80 phycoerythrin (PE) and anti- CD1a-FITC. Staining was performed for 30 min at 4 °C, and cells were washed twice in staining buffer and resuspended in PBS.

To determine Treg phenotype, cultured CD4 + lymphocytes were first extracellularly stained with CD45RO-APC, CD25-FICT and CD127 PE-Cy7 (eBiosciences, San Diego, CA). Then, cells were fixed, permeabilized and intracellularly stained with anti-FOXP3 PE (clone PCH101) following the manufacturer’s instructions (eBiosciences). Isotype controls were used to set up quadrants and CD4 + CD25+T cells were subdivided into CD25low and CD25high populations. A minimum of 10,000 cells were acquired and analyzed using the FACS Diva Software 6.1.2 (BD Biosciences). The specific fluorescence intensity was quantified as the mean fluorescence intensity (MFI) calculated by subtracting the background of isotype matched control staining from the total fluorescence.

### Statistical analysis

Data were expressed as mean ± standard deviation (SD) from three independent experimental runs performed using separate cryopreserved aliquots from the same pooled PBMC preparation. Technical replicates within each experiment were averaged before statistical analysis. One-way ANOVA followed by Tukey’s post hoc test was used for comparisons among experimental groups. A significance level of 0.05 was established for all tests, and data analysis was performed using GraphPad Prism software, version 10.0.

## Results

### Dendritic cell viability

**Fig. 1:**
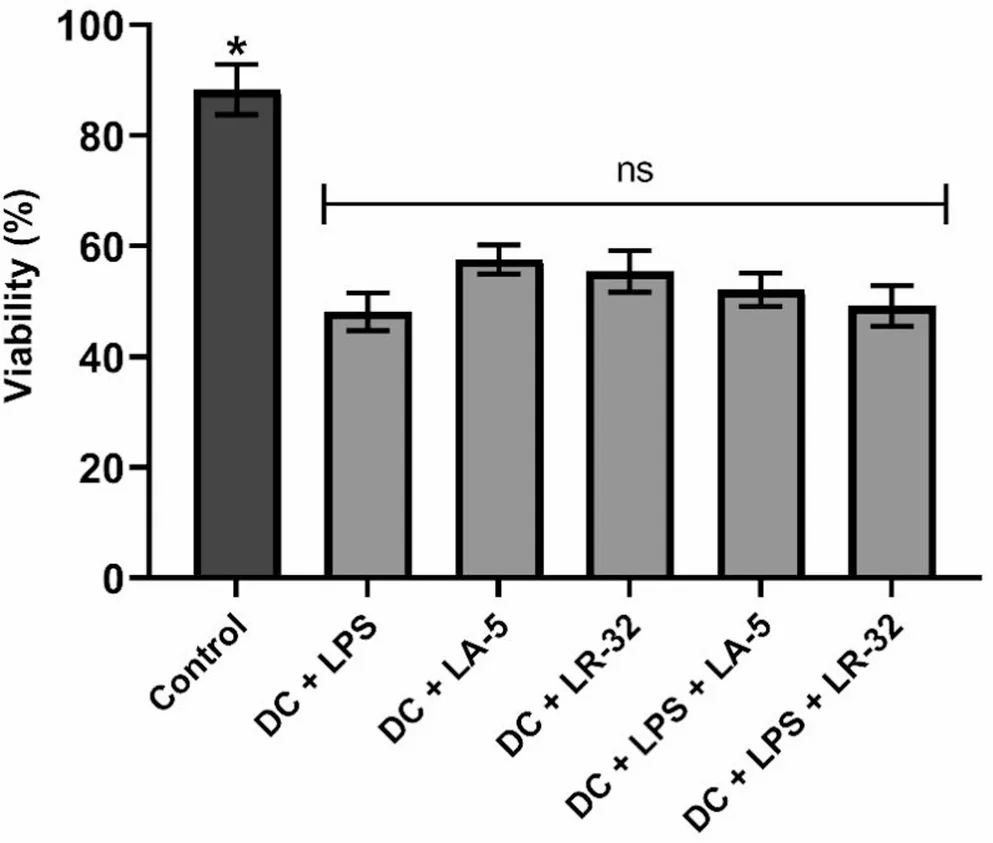
Cell viability (%) of untreated DCs (control), DCs treated with *E. coli* LPS (DC + LPS), *L. acidophilus* LA-5 (DC + LA) or *L. rahmnosus* LR-32 (DC + LR) determined by the LDH cytotoxicity assay. The asterisk indicates statistical significance between the control group and the other groups (*p* < 0.05), and “ns” indicates no statistical difference among the groups (*p* > 0.05)

Exposure of dendritic cells (DCs) to LPS or probiotic strains resulted in reduced cell viability compared with the untreated control group (*p* < 0.05). However, no statistically significant differences were observed among the stimulated experimental conditions, including DCs treated with LPS, LA-5, LR-32, or the combinations of LPS with each probiotic strain (Fig. 1). Thus, although stimulation affected overall viability, the different immunological responses observed among the stimulated groups were not associated with significant differences in cell viability.

### Probiotics modulate pattern-recognition receptor gene expression in immature and LPS-stimulated DCs

**Fig. 2:**
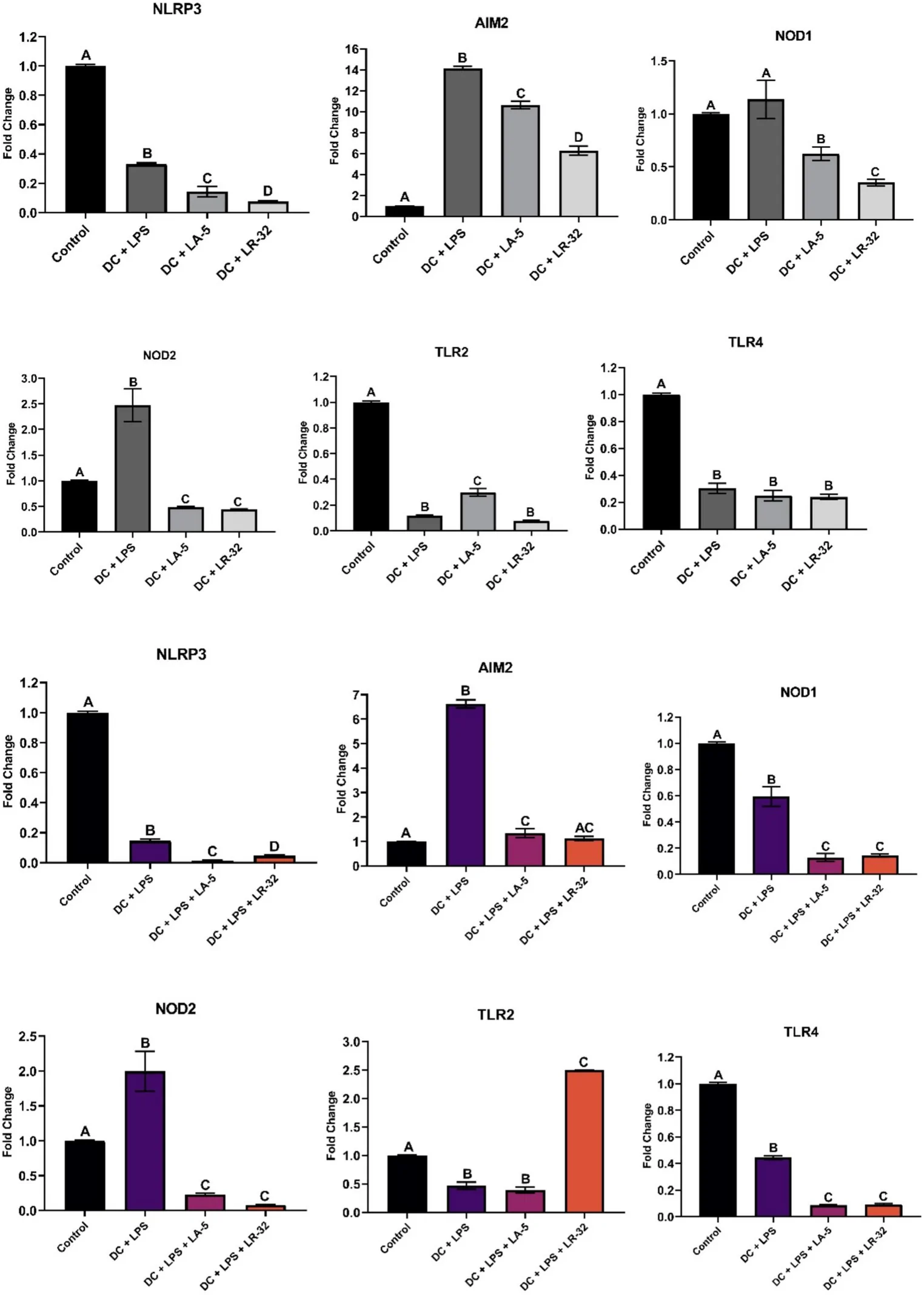
Pattern-recognition receptor gene expression in DCs exposed to LPS, probiotics, and LPS/probiotics. Relative gene expression levels of NLRP3, AIM2, NOD1, NOD2, TLR2, and TLR4 in untreated dendritic cells (DCs; control), DCs stimulated with *E. coli* LPS (DC + LPS), DCs exposed to *L. acidophilus* LA-5 (DC + LA-5) or *L. rhamnosus* LR-32 (DC + LR-32), and DCs co-stimulated with LPS plus LA-5 (DC + LPS+LA-5) or LPS plus LR-32 (DC + LPS+LR-32). Gene expression was calculated using the ΔΔCt method, with GAPDH as the endogenous control, and expressed as mean fold change ± SD. Different letters indicate statistically significant differences among groups within the same gene/panel according to one-way ANOVA followed by Tukey’s post hoc test (*p* < 0.05). Groups sharing the same letter are not significantly different

The gene expression analysis showed that LPS stimulation modulated the transcription of genes encoding pattern-recognition receptors in DCs. In general, LPS reduced the expression of most receptor-related genes, with the exception of NOD1, which remained upregulated compared with untreated DCs (Fig. 2).

Exposure to probiotic strains alone also reduced the expression of most receptor-related genes. This effect was strain-dependent, since *Lacticaseibacillus rhamnosus* LR-32 induced a more pronounced suppression of NLRP3, NOD1, TLR2, and TLR4 gene expression compared with *Lactobacillus acidophilus* LA-5 (*p* < 0.001). In contrast, AIM2 expression was increased by LPS and remained higher in DCs exposed to LA-5 and LR-32 than in untreated controls, although both probiotic strains induced lower AIM2 expression than LPS alone (Fig. 2).

When DCs were co-stimulated with LPS and probiotic strains, a general downregulation of receptor-related genes was observed compared with LPS-stimulated DCs. This effect was evident for NLRP3, NOD1, NOD2, TLR4, and AIM2 (Fig. 2). However, LR-32 showed a distinct effect on TLR2 expression under LPS-stimulated conditions, increasing TLR2 transcription compared with both LPS alone and LPS combined with LA-5. These findings indicate that LA-5 and LR-32 differentially modulate pattern-recognition receptor gene expression in both immature and LPS-stimulated DCs.

### Probiotics differentially regulate inflammatory and regulatory cytokine gene expression

**Fig. 3:**
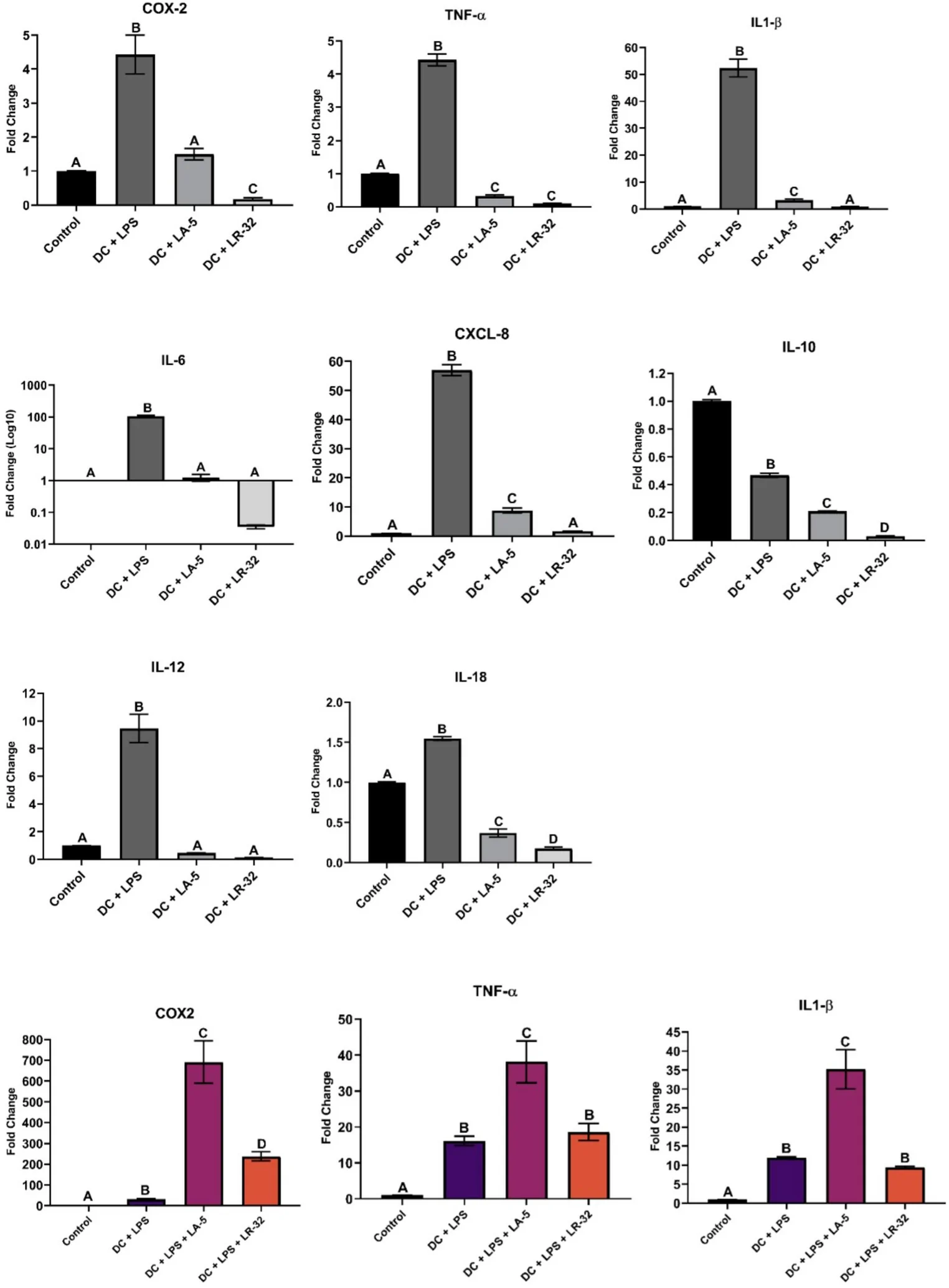

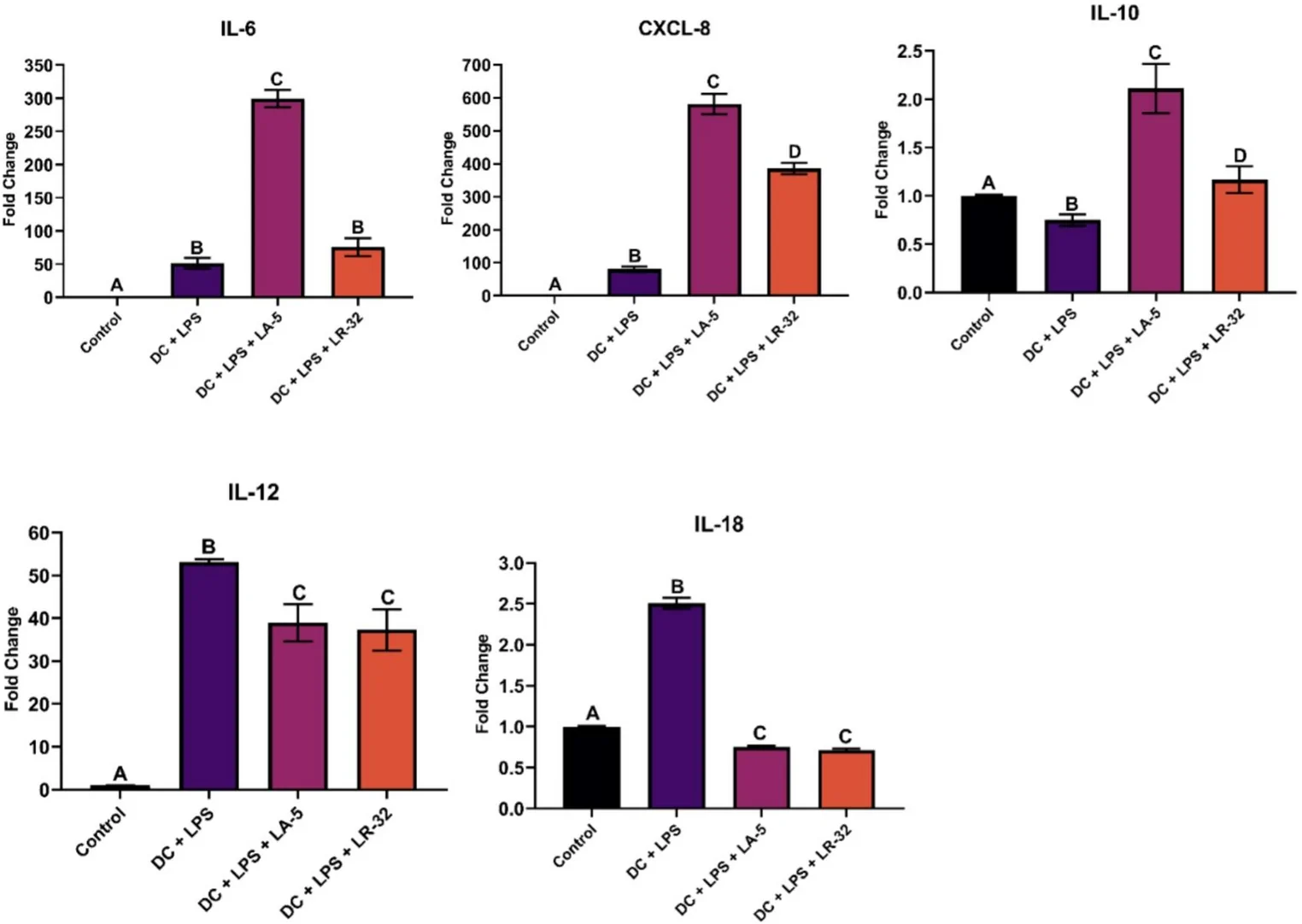
Inflammatory and regulatory mediator gene expression in DCs exposed to LPS, probiotics, and LPS/probiotics. Relative gene expression levels of COX-2, TNF-α, IL-1β, IL-6, CXCL-8, IL-10, IL-12, and IL-18 in untreated dendritic cells (DCs; control), DCs stimulated with *E. coli* LPS (DC + LPS), DCs exposed to *L. acidophilus* LA-5 (DC + LA-5) or *L. rhamnosus* LR-32 (DC + LR-32), and DCs co-stimulated with LPS plus LA-5 (DC + LPS+LA-5) or LPS plus LR-32 (DC + LPS+LR-32). Gene expression was calculated using the ΔΔCt method, with GAPDH as the endogenous control, and expressed as mean fold change ± SD. Different letters indicate statistically significant differences among groups within the same gene/panel according to one-way ANOVA followed by Tukey’s post hoc test (*p* < 0.05). Groups sharing the same letter are not significantly different

LPS stimulation significantly increased the transcription of inflammatory mediators, including COX-2, TNF-α, IL-1β, IL-6, CXCL-8, IL-12, and IL-18, compared with untreated DCs (*p* < 0.001), indicating induction of an inflammatory activation profile (Figs. 3). In contrast, stimulation with probiotic strains alone did not induce a comparable inflammatory transcriptional profile, since most mRNA levels remained at or below those observed in untreated DCs. An exception was observed for CXCL-8, which was upregulated in DCs exposed to LA-5 (Fig. 3).

The presence of probiotics modified the LPS-induced inflammatory response. Co-stimulation with LA-5 increased the transcription of TNF-α, IL-1β, and IL-6 compared with both LPS alone and LPS combined with LR-32 (*p* < 0.05). In contrast, although LR-32 combined with LPS induced lower COX-2 and CXCL-8 gene expression than LA-5 combined with LPS, these levels remained significantly higher than those observed in DCs stimulated with LPS alone (Fig. 3; *p* < 0.05).

Regulatory cytokine gene expression was also affected by the experimental conditions. LPS stimulation reduced IL-10 transcription compared with untreated DCs, and this effect was further intensified by exposure to probiotic strains alone (*p* < 0.001). However, under LPS-stimulated conditions, LA-5 increased IL-10 gene expression compared with both LPS alone and LPS combined with LR-32 (*p* < 0.05). For IL-12 and IL-18, co-stimulation with probiotic strains resulted in intermediate expression levels, which were lower than those induced by LPS alone but higher than those observed in untreated DCs (Fig. 3; *p* < 0.05). Together, these data indicate that LA-5 and LR-32 differentially regulate inflammatory and regulatory cytokine gene expression in DCs, particularly under LPS-induced stimulation.

### Cytokine secretion after LPS and probiotic stimulation

Cytokine quantification in culture supernatants confirmed that LPS significantly increased the production of inflammatory mediators compared with untreated DCs and probiotic-only conditions (*p* < 0.05; Table 1). Co-treatment with LA-5 did not significantly alter TNF-α or IL-6 levels compared with LPS alone. However, both probiotic strains significantly reduced CXCL-8 production in LPS-stimulated DCs (*p* < 0.05).

**Table 1:** Mean (± SD) of cytokines levels (pg/mL) in the culture media supernatant of untreated DCs (control), DCs treated with *E. coli* LPS (DC + LPS), or with probiotics *L. acidophilus* LA-5 (DC + LA-5) or L. rahmnosus LR-32 (DC + LR-32), and DCs treated with LPS and *L. acidophilus* LA-5 (DC + LPS+LA-5) or *L. rahmnosus* LR-32 (DC + LPS+LR-32)

Cytokines	Experimental groups					
	CONTROL	DC + LA-5	DC + LR-32	DC + LPS	DC + LPS+LA-5	DC + LPS+LR-32
TNF-α	11.8 (4.04)^A^	13.44 (0.37)^A^	17.11 (2.66)^A^	381.23 (89.08)^B^	430.22 (53.46)^B^	122.33 (18.31)^C^
IL1-β	0.87 (0.10)^A^	1.78 (0.11)^B^	1.18 (0.26)^A^	1.02 (0.27)^A^	1.93 (0.26)^B^	0.96 (0.02)^A^
IL-6	6.75 (0.69)^A^	6.54 (0.47)^A^	6.52 (0.13)^A^	310.98 (11.85)^B^	282.21 (12.05)^B^	165.76 (76.62)^C^
CXCL-8	17.34 (7.55)^A^	35.28 (2.12)^A^	83.8 (22.89)^B^	496.90 (20.10)^C^	223.28 (4.52)^D^	225.15 (7.96)^D^

Different letters indicate statistically significant differences among groups within the lines according to one-way ANOVA followed by Tukey’s post hoc test (*p* < 0.001)

These results indicate that changes observed at the transcriptional level were only partially reflected at the protein level. Nevertheless, the reduction in CXCL-8 secretion suggests that both LA-5 and LR-32 can attenuate selected components of the LPS-induced inflammatory response in DCs.

### Flow cytometric analysis indicates partial DC activation/maturation

**Fig. 4:**
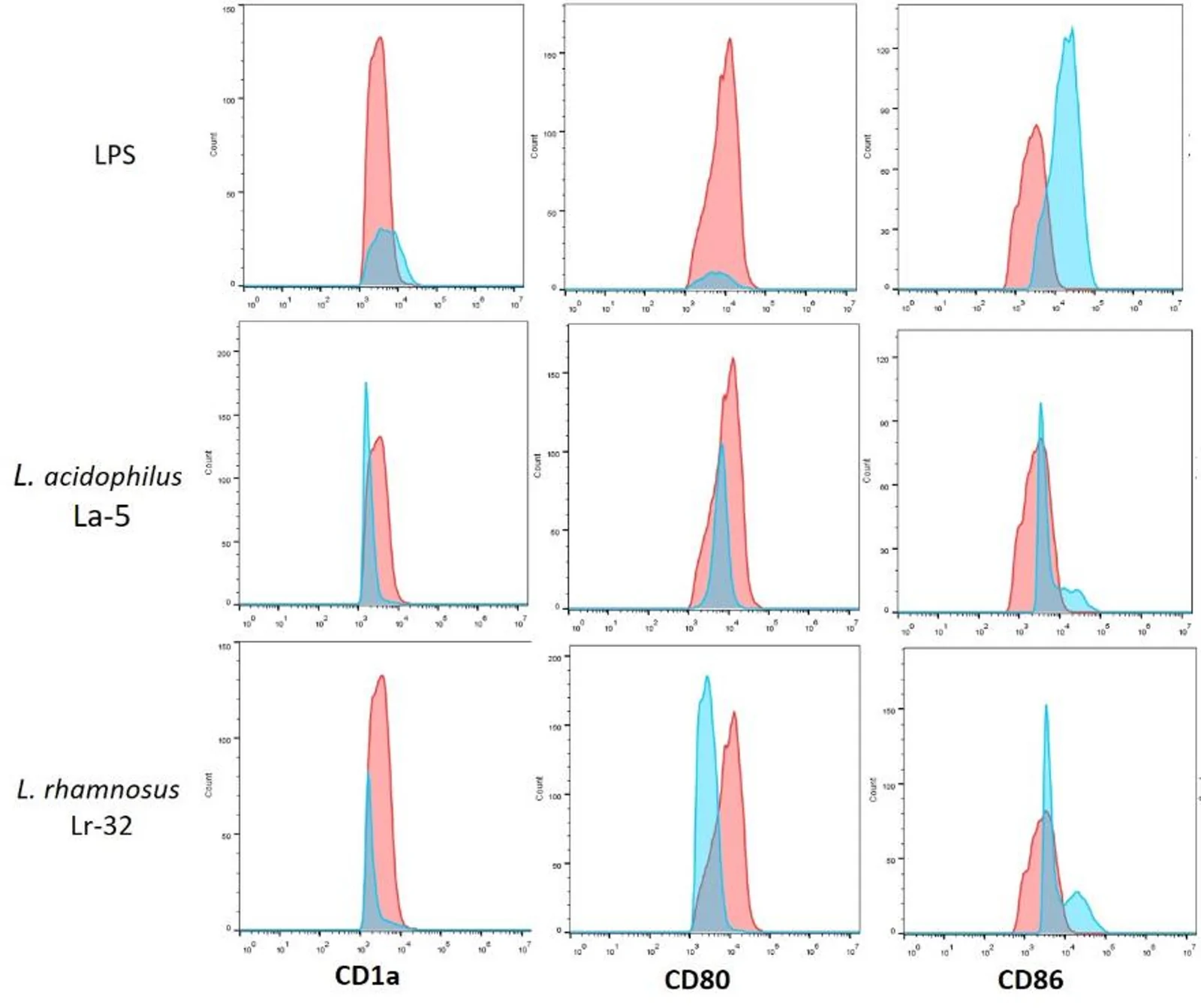
Maturation of monocyte-derived DC treated with probiotic Lactobacilli strains. Immature DC were cultured for 24 h with (LPS) or with different lactobacilli strains at a bacteria: cell ratio of 10:1. Expression of CD1a, CD80, CD86 was analyzed by flow cytometry. Representative profile after 24 h of culture with LPS or lactobacilli strains (*L. acidophillus* LA-5 or *L. rhamnosus* LR-32). Red histograms belong to immature DCs and blue histograms represent specific staining of the indicated cell surface marker in LPS or Lactobacilli-stimulated DC

Flow cytometric analysis demonstrated that exposure to LPS and probiotic strains altered the expression of surface markers associated with DC activation. Both probiotic strains increased the expression of the co-stimulatory molecules CD80 and CD86 and reduced CD1a expression compared with untreated DCs (Fig. 4).

The changes induced by LA-5 and LR-32 were comparable to those observed after LPS stimulation for some markers. However, because CD86 expression was more consistently modulated than CD80, these findings are better interpreted as evidence of partial DC activation or modulation of the co-stimulatory phenotype rather than complete DC maturation. Thus, probiotic exposure altered the phenotypic profile of DCs, but the magnitude and pattern of this modulation depended on the marker evaluated and on the probiotic strain.

### DCs exposed to probiotics modulate a Treg-associated phenotype in CD4⁺ T cells

**Fig. 5:**
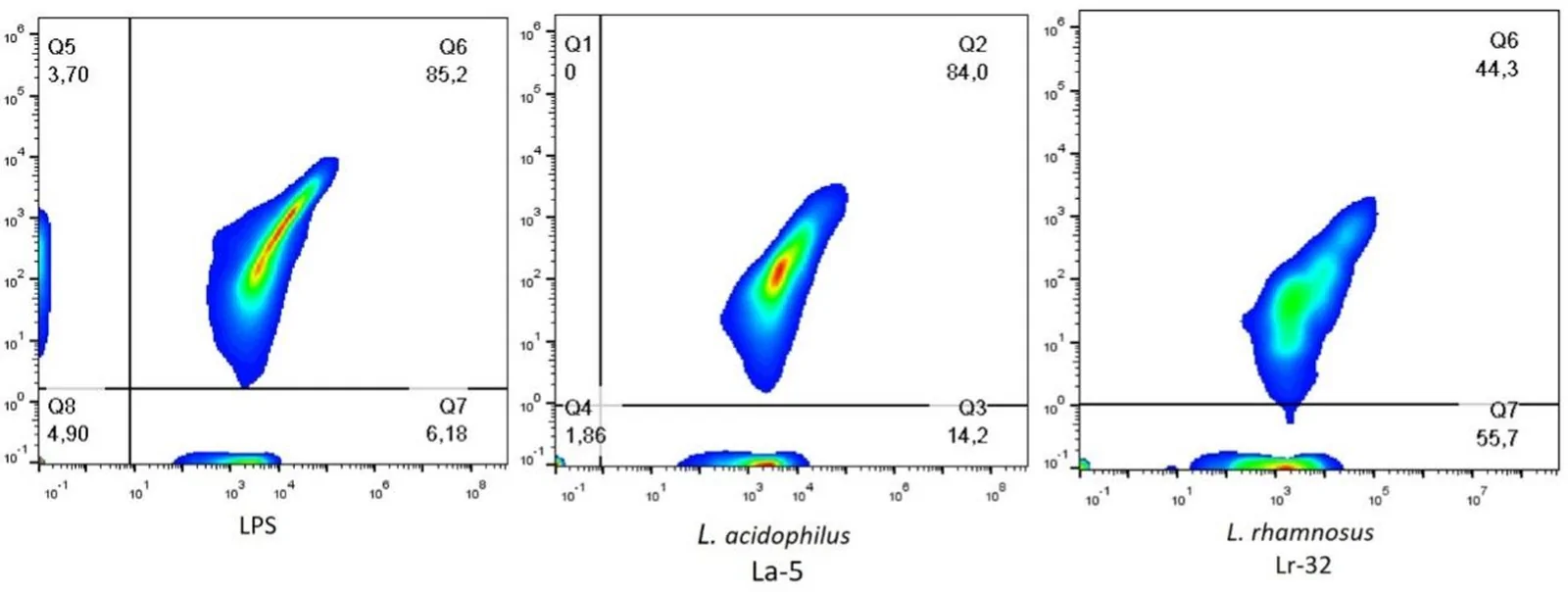
Representative flow cytometry plots showing the gating strategy used to identify Treg-associated cells after DC–T cell co-culture. Lymphocytes were first gated according to FSC/SSC parameters, followed by selection of CD45RO⁺ cells. Within this population, CD25high cells were analyzed for FOXP3high and CD127low expression. Gates were defined using isotype/FMO controls and applied consistently across all groups. The percentage of CD25highFOXP3highCD127low cells was used to define the Treg-associated phenotype

The functional impact of probiotic-exposed DCs was evaluated in DC–T cell co-cultures. DCs treated with LA-5 induced phenotypic changes in CD4⁺ T lymphocytes consistent with a Treg-associated profile, as indicated by increased frequencies of CD25highFOXP3highCD127low cells (Fig. 5). This effect was comparable to that observed with LPS-treated DCs.

In contrast, DCs stimulated with LR-32 induced a lower proportion of CD25highFOXP3highCD127low cells than LA-5-treated DCs. These results suggest that the probiotic strains differ in their capacity to modulate DC-driven CD4⁺ T cell responses. In particular, LA-5-treated DCs showed a greater ability to promote a Treg-associated phenotype under the conditions evaluated in this study.

**Fig. 6:**
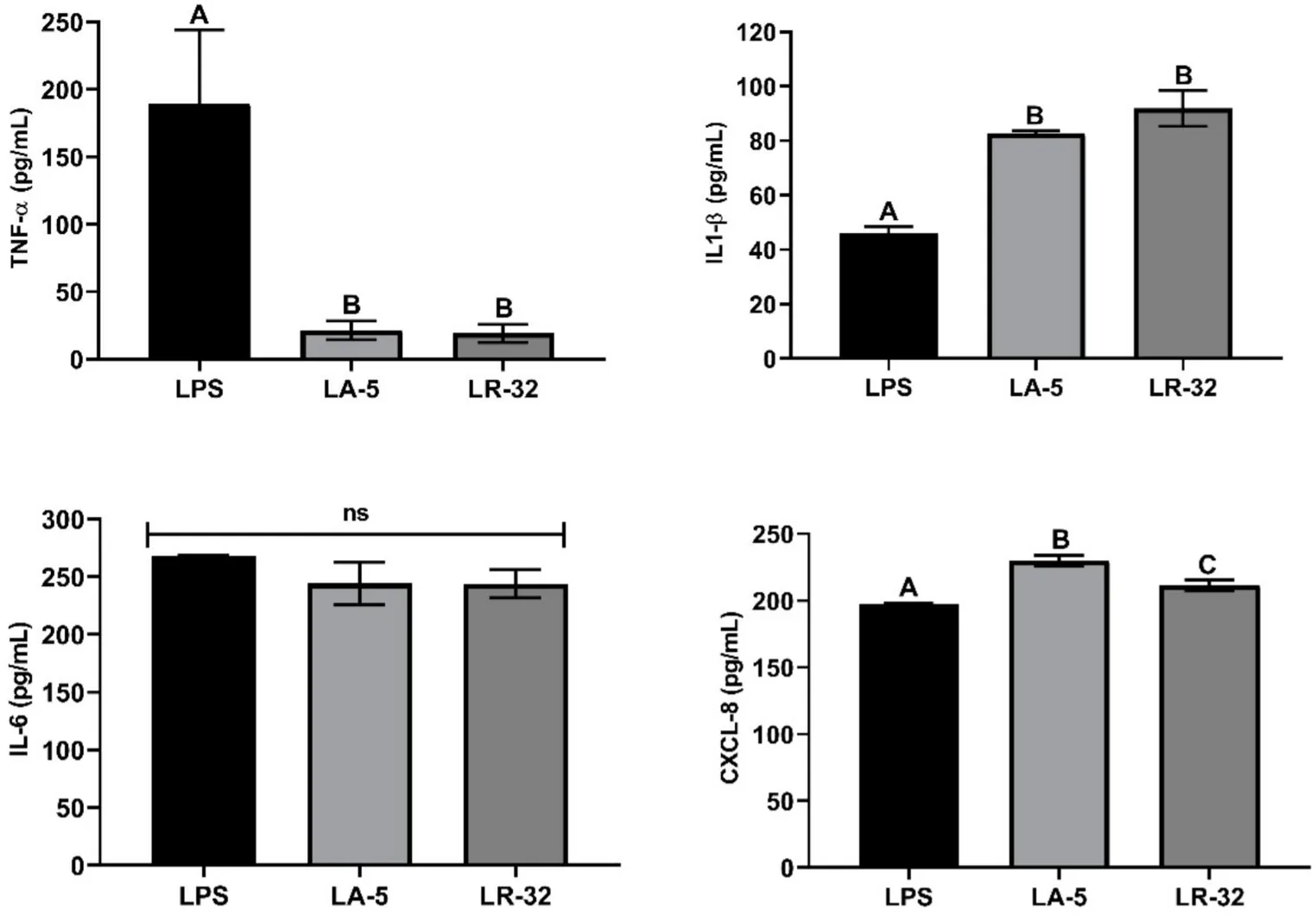
Cytokine production (pg/mL) by CD4 + T lymphocytes stimulated with probiotics treated DCs. Immature DCs were treated with LPS, *L. acidophilus* LA-5 or *L. rahmnosus* LR-32 were cocultured with allogeneic naive CD4 + CD45RA + T cells. The concentration of TNF-α, IL1-β, IL-6 and CXCL-8 was quantified by ELISA. Vertical bars denote SD. Different letters indicate statistically significant differences among groups within the same panel according to one-way ANOVA followed by Tukey’s post hoc test (*p* < 0.05). “ns” indicate no statistical difference among the groups (*p* > 0.05)

The soluble inflammatory profile of DC–T cell co-cultures was also evaluated after stimulation of immature DCs with LPS, LA-5, or LR-32 followed by co-culture with allogeneic naïve CD4⁺CD45RA⁺ T cells. TNF-α levels were higher in co-cultures containing LPS-treated DCs than in those containing LA-5- or LR-32-treated DCs (*p* < 0.05). In contrast, IL-1β production was increased in co-cultures with probiotic-treated DCs compared with LPS-treated DCs, with no significant difference between LA-5 and LR-32 (*p* < 0.05). IL-6 levels did not differ significantly among the groups (*p* > 0.05). CXCL-8 production differed among all experimental conditions, with higher levels observed in co-cultures with LA-5-treated DCs, followed by LR-32-treated DCs and LPS-treated DCs (*p* < 0.05; Fig. 6). These findings indicate that probiotic-treated DCs modified the soluble inflammatory milieu of DC–T cell co-cultures in a cytokine-dependent and strain-dependent manner.

## Discussion

The present study showed that *L. acidophilus* LA-5 and *L. rhamnosus* LR-32 modulate human monocyte-derived dendritic cell responses in a strain-dependent manner. Rather than acting as simple anti-inflammatory stimuli, these probiotic strains reshaped DC activation by altering pattern-recognition receptor gene expression, cytokine transcription and secretion, co-stimulatory molecule expression, and the ability of DCs to induce a Treg-associated phenotype in CD4⁺ T cells. Since the functional ability of DCs depends on the integration of receptor signaling, cytokine production, co-stimulatory molecule expression, and cell survival, the data suggest that LA-5 and LR-32 induce distinct immunological programs in DCs (Kaliński et al. 2000; Pulendran et al. 2001; Kapsenberg 2003). This reinforces the concept that probiotic immunomodulation is highly strain-dependent and influenced by the inflammatory context in which host cells encounter bacteria (Smits et al. 2005; Foligne et al. 2007).

Cell viability was reduced in all stimulated groups compared with untreated DCs. However, no significant differences were observed among DCs exposed to LPS, probiotics, or the combinations of LPS with each probiotic strain. Therefore, although the reduction in viability should be considered when interpreting the magnitude of cytokine production and gene expression, the differences observed among stimulated groups are unlikely to be explained only by differential cytotoxicity. This point is relevant because live bacteria and LPS may impose metabolic and inflammatory stress on DCs during 24 h of stimulation. Thus, the viability data support cautious interpretation of the results, while also indicating that the strain-specific effects occurred under comparable viability conditions.

LPS stimulation induced broad modulation of genes encoding extracellular and intracellular pattern-recognition receptors, with downregulation of several receptors and upregulation of NOD1. Although NOD1 is classically recognized as a sensor of peptidoglycan-derived motifs containing meso-diaminopimelic acid, it may also participate in the amplification of inflammatory responses and in the regulation of cytokine signaling in human DCs (Chamaillard et al. 2003; Neuper et al. 2017; Bi et al. 2018). Therefore, the maintenance or increase of NOD1 expression after LPS exposure may reflect its participation in downstream inflammatory signaling rather than only canonical peptidoglycan recognition. This result suggests that DC activation by LPS involves not only membrane TLR signaling but also intracellular regulatory pathways that contribute to the inflammatory profile.

The reduction in TLR transcript levels after LPS or probiotic exposure should not be interpreted as absence of activation. TLR signaling is highly dynamic, and receptor downregulation may occur after ligand engagement as part of feedback mechanisms that prevent excessive inflammatory responses (Peroval et al. 2013). In this study, decreased TLR4 expression after LPS stimulation occurred together with increased transcription of inflammatory mediators such as COX-2, TNF-α, IL-1β, IL-6, and CXCL-8. This apparent dissociation between receptor transcript levels and downstream inflammatory mediators is compatible with receptor engagement followed by regulatory attenuation of receptor expression. Similar mechanisms have been reported for lactobacilli, which can modulate TLR2/TLR4-related responses and induce regulatory pathways in antigen-presenting cells in a strain-dependent manner (Kaji et al. 2010; Fong et al. 2015; Mikulic et al. 2017).

Both probiotic strains modulated receptor gene expression in immature and LPS-stimulated DCs. This effect was more pronounced for LR-32, which reduced the expression of NLRP3, NOD1, TLR2, and TLR4 in DCs exposed to probiotics alone. In LPS-stimulated DCs, probiotic exposure also reduced several receptor-related genes compared with LPS alone, indicating that LA-5 and LR-32 can interfere with inflammatory receptor signaling. However, LR-32 increased TLR2 expression in the presence of LPS, which may be related to the recognition of lactobacilli-derived components, such as lipoteichoic acids and other cell wall structures, by TLR2 (Mohamadzadeh et al. 2005; Ciorba et al. 2012). Taken together, these findings indicate that probiotic–DC interaction is not uniform and depends on both the strain and the activation state of DCs.

The DC ratio of 1:10 was selected because previous in vitro studies with lactobacilli and antigen-presenting cells, including studies using LA-5 and LR-32, showed that this ratio enables detectable immune modulation while limiting excessive bacterial overload. Nevertheless, this ratio represents a simplified in vitro condition and does not reproduce the microbial density, spatial organization, or polymicrobial complexity of periodontal biofilms. Therefore, the present findings should be interpreted as evidence of controlled probiotic modulation of DC responses rather than as a direct simulation of the periodontal microenvironment.

The cytokine gene expression profile further demonstrated that LA-5 and LR-32 exert distinct immunomodulatory effects. LPS strongly increased inflammatory mediator transcription, as expected for a classical TLR4-mediated inflammatory stimulus. In contrast, probiotics alone induced a weaker inflammatory transcriptional profile, with most genes remaining at or below basal levels, except for CXCL-8 in LA-5-treated DCs. Under LPS-stimulated conditions, LA-5 increased TNF-α, IL-1β, and IL-6 transcription compared with LPS alone, whereas LR-32 showed a more suppressive profile for selected inflammatory mediators. This indicates that LA-5 and LR-32 do not simply inhibit LPS-induced activation; instead, they redirect the inflammatory response in different ways.

The increased transcription of TNF-α, IL-6, and IL-1β induced by LA-5 should not be interpreted as incompatible with the Treg-associated phenotype observed in the DC–T cell co-culture. DCs can acquire mixed activation states in which inflammatory signaling coexists with regulatory programming. Thus, the Treg-associated phenotype likely reflects the integrated DC signal, including co-stimulatory molecule expression, regulatory cytokine pathways, receptor engagement, and the temporal sequence of activation, rather than the isolated effect of a single cytokine. In this context, LA-5 appears to induce a controlled activation profile, not a purely pro-inflammatory or purely tolerogenic phenotype.

IL-10 and IL-12 are central regulators of DC-driven immune responses. IL-12 favors protective Th1-associated immunity, whereas IL-10 limits excessive inflammation and contributes to immune regulation (Trinchieri 2003; Ouyang et al. 2011). Their production is reciprocally regulated depending on the cellular context and microbial stimulus (Ma et al. 2015). In the present study, LA-5 increased IL-10 gene expression under LPS-stimulated conditions compared with LPS alone or LPS combined with LR-32, while IL-12 and IL-18 expression were lower than in DCs stimulated only with LPS. This suggests that LA-5 may preserve selected inflammatory signals while attenuating others and favoring a regulatory component in activated DCs.

The relationship between gene expression and cytokine secretion was only partial. Although LPS increased inflammatory mediator transcription, ELISA data showed that probiotic co-treatment did not uniformly reproduce the transcriptional changes at the protein level. Both probiotic strains reduced CXCL-8 secretion in LPS-stimulated DCs, whereas TNF-α and IL-6 were not significantly altered by LA-5 compared with LPS alone. This discrepancy may reflect post-transcriptional regulation, different kinetics between mRNA accumulation and protein secretion, or the influence of cell viability and bacterial interaction during the 24 h culture period. Therefore, the cytokine data indicate selective attenuation of the LPS-induced secretory response, particularly for CXCL-8, rather than global suppression of inflammation.

The flow cytometry results indicate that probiotic exposure altered the DC co-stimulatory phenotype. Both strains increased the expression of co-stimulatory molecules and reduced CD1a expression compared with untreated DCs. However, because CD86 was more consistently modulated than CD80, these findings are better interpreted as partial DC activation or modulation of the co-stimulatory profile rather than complete DC maturation. This distinction is important because full DC maturation requires coordinated changes in surface markers, cytokine production, antigen-presenting capacity, and functional T cell polarization. Thus, LA-5 and LR-32 appear to modulate DC activation status without necessarily inducing a fully mature phenotype identical to that induced by LPS.

Day 7 was selected based on standard protocols for generating monocyte-derived immature DCs using GM-CSF and IL-4. The untreated day-7 DC group served as the baseline control for comparison with LPS- and probiotic-treated groups. However, basal activation cannot be completely excluded in long-term in vitro cultures. For this reason, the results should be interpreted relative to the untreated day-7 control rather than as evidence that these cells were fully resting or completely non-activated. This clarification is particularly relevant when interpreting changes in receptor expression and co-stimulatory markers after probiotic exposure.

The DC–T cell co-culture results suggest that LA-5-exposed DCs favored the emergence of a CD25highFOXP3highCD127low phenotype in CD4⁺ T cells. This phenotype is consistent with a Treg-associated profile, especially because FOXP3 was evaluated after 12 days of culture, a time point beyond the transient FOXP3 upregulation commonly observed after conventional T cell activation (Tran et al. 2007; Pillai et al. 2007; Fontenot et al. 2017). These findings are consistent with previous studies showing that probiotics can promote regulatory DCs and CD4⁺FOXP3⁺ T cell populations in inflammatory models (Kwon et al. 2010; Lavasani et al. 2010). Nevertheless, functional suppression assays would be required to confirm that these cells exert bona fide regulatory activity.

The cytokines measured in DC–T cell co-culture supernatants should be interpreted as the soluble inflammatory milieu of the co-culture system, not as definitive markers of T helper differentiation. TNF-α, IL-1β, IL-6, and CXCL-8 are relevant inflammatory mediators, but they are not canonical markers of Th1, Th2, Th17, or Treg polarization. In addition, ELISA measurements in mixed DC–T cell cultures do not identify the cellular source of each cytokine. Therefore, these data cannot distinguish whether cytokines were produced predominantly by DCs, T cells, or both. Future studies should include intracellular cytokine staining, cell sorting before cytokine analysis, or canonical T cell polarization markers such as IFN-γ, IL-4, IL-17, IL-10, and TGF-β.

Although the study was framed within the context of periodontal immunomodulation, the experimental model used *E. coli* LPS as a standardized TLR4-dependent stimulus. This approach allowed controlled assessment of probiotic effects on inflammatory DC activation but does not reproduce the complexity of periodontal pathobionts, such as *P. gingivalis*, or polymicrobial oral biofilms. *P. gingivalis* LPS and other virulence factors may activate DCs through distinct pathways, including TLR2-biased signaling and immune subversion mechanisms (Kanaya et al. 2009). Therefore, direct extrapolation to periodontal disease should be made cautiously. Future studies should evaluate LA-5 and LR-32 in DC models challenged with oral pathogen-derived LPS, whole periodontal bacteria, or multispecies biofilms.

This study has additional limitations. Because PBMCs from all donors were pooled before cryopreservation and experimental stimulation, inter-donor variability was not directly assessed; therefore, donor-specific immunomodulatory patterns cannot be inferred from the present data. The three independent experimental runs were performed using separate cryopreserved aliquots from the same pooled PBMC preparation, which should be considered when interpreting the statistical analysis. In addition, the use of a single MOI and one main stimulation time point limits the evaluation of dose- and time-dependent probiotic effects. Moreover, the study did not include intracellular cytokine staining, functional Treg suppression assays, or canonical T helper cytokine panels. Despite these limitations, the combined analysis of gene expression, cytokine secretion, surface markers, and DC–T cell co-culture provides evidence that LA-5 and LR-32 differentially modulate DC responses.

In summary, LA-5 and LR-32 modulated human DC responses in a strain-dependent manner. LR-32 showed a stronger suppressive effect on several pattern-recognition receptor genes, whereas LA-5 induced a mixed activation profile characterized by selected inflammatory mediator expression, increased IL-10 transcription under LPS stimulation, modulation of co-stimulatory markers, and greater induction of a Treg-associated CD4⁺ T cell phenotype. Thus, these probiotic strains appear to reshape, rather than simply suppress, DC activation. Such modulation may be relevant for mucosal immune regulation, but further studies using periodontal pathobionts and functional T cell assays are required to define their potential role in controlling periodontal inflammation.

## Supplementary Information

Below is the link to the electronic supplementary material.

Supplementary Material 1

## Data Availability

All data generated or analyzed during this study are included in this article. Further inquiries can be directed to the corresponding author.
