# Supplementary material for: Immunomodulatory effect of Lacticaseibacillus rhamnosus and Lactobacillus acidophilus in human dendritic cells
Source: Arch Microbiol. 2026 Aug 10;208(11):555. doi: 10.1007/s00203-026-05135-7 (PMC13457254; doi:10.1007/s00203-026-05135-7)
Supplement: Supplementary file 1 [file 203_2026_5135_MOESM1_ESM.docx]

Supplementary Table 1. TaqMan assays used for RT-qPCR analysis.

| Target | Gene symbol | Manufacturer | Endogenous control | Platform |
| --- | --- | --- | --- | --- |
| NLR family pyrin domain containing 3 | NLRP3 | Applied Biosystems/Thermo Fisher Scientific | GAPDH | TaqMan RT-qPCR |
| Absent in melanoma 2 | AIM2 | Applied Biosystems/Thermo Fisher Scientific | GAPDH | TaqMan RT-qPCR |
| Nucleotide-binding oligomerization domain containing 1 | NOD1 | Applied Biosystems/Thermo Fisher Scientific | GAPDH | TaqMan RT-qPCR |
| Nucleotide-binding oligomerization domain containing 2 | NOD2 | Applied Biosystems/Thermo Fisher Scientific | GAPDH | TaqMan RT-qPCR |
| Toll-like receptor 2 | TLR2 | Applied Biosystems/Thermo Fisher Scientific | GAPDH | TaqMan RT-qPCR |
| Toll-like receptor 4 | TLR4 | Applied Biosystems/Thermo Fisher Scientific | GAPDH | TaqMan RT-qPCR |
| Cyclooxygenase-2 / Prostaglandin-endoperoxide synthase 2 | PTGS2 | Applied Biosystems/Thermo Fisher Scientific | GAPDH | TaqMan RT-qPCR |
| Tumor necrosis factor alpha | TNF | Applied Biosystems/Thermo Fisher Scientific | GAPDH | TaqMan RT-qPCR |
| Interleukin-1 beta | IL1B | Applied Biosystems/Thermo Fisher Scientific | GAPDH | TaqMan RT-qPCR |
| Interleukin-6 | IL6 | Applied Biosystems/Thermo Fisher Scientific | GAPDH | TaqMan RT-qPCR |
| C-X-C motif chemokine ligand 8 / Interleukin-8 | CXCL8 | Applied Biosystems/Thermo Fisher Scientific | GAPDH | TaqMan RT-qPCR |
| Interleukin-10 | IL10 | Applied Biosystems/Thermo Fisher Scientific | GAPDH | TaqMan RT-qPCR |
| Interleukin-12 | IL12A | Applied Biosystems/Thermo Fisher Scientific | GAPDH | TaqMan RT-qPCR |
| Interleukin-18 | IL18 | Applied Biosystems/Thermo Fisher Scientific | GAPDH | TaqMan RT-qPCR |
| Glyceraldehyde-3-phosphate dehydrogenase | GAPDH | Applied Biosystems/Thermo Fisher Scientific | Endogenous control | TaqMan RT-qPCR |

Supplementary Table 2. Antibodies used for flow cytometry analysis.

| Target | Fluorochrome | Supplier | Cell population | Purpose |
| --- | --- | --- | --- | --- |
| CD86 | FITC | Miltenyi Biotech | DCs | Co-stimulatory molecule |
| CD80 | PE | Miltenyi Biotech | DCs | Co-stimulatory molecule |
| CD1a | FITC | Miltenyi Biotech | DCs | DC phenotype marker |
| CD45RO | APC | eBioscience | T cells | Memory/activated T cell phenotype |
| CD25 | FITC | eBioscience | T cells | Treg-associated marker |
| CD127 | PE-Cy7 | eBioscience | T cells | Treg-associated marker |
| FOXP3 | PE | eBioscience | T cells | Intracellular Treg marker |
